# Prevention of 5-hydroxytryptamine_2C_ receptor RNA editing and alternate splicing in C57BL/6 mice activates the hypothalamic-pituitary-adrenal axis and alters mood

**DOI:** 10.1111/ejn.12727

**Published:** 2014-09-26

**Authors:** Vincent Bombail, Wei Qing, Karen E Chapman, Megan C Holmes

**Affiliations:** University/BHF Centre for Cardiovascular Science, The Queen's Medical Research Institute, University of EdinburghEdinburgh, UK; * INRA-UR1197, Neurobiologie de l'Olfaction (NBO)Domaine de Vilvert, 78350, Jouy-en-Josas, France

**Keywords:** anxiety, depression, serotonin, stress

## Abstract

The 5-hydroxytryptamine_2C_ (5-HT)_2C_ receptor is widely implicated in the aetiology of affective and eating disorders as well as regulation of the hypothalamo-pituitary-adrenal axis. Signalling through this receptor is regulated by A-to-I RNA editing, affecting three amino acids in the protein sequence, with unedited transcripts encoding a receptor (INI) that, *in vitro*, is hyperactive compared with edited isoforms. Targeted alteration (knock-in) of the *Htr2c* gene to generate ‘INI’ mice with no alternate splicing, solely expressing the full-length unedited isoform, did not produce an overt metabolic phenotype or altered anxiety behaviour, but did display reduced depressive-like and fear-associated behaviours. INI mice exhibited a hyperactive hypothalamo-pituitary-adrenal axis, with increased nadir plasma corticosterone and corticotrophin-releasing hormone expression in the hypothalamus but responded normally to chronic stress and showed normal circadian activity and activity in a novel environment. The circadian patterns of 5-HT_2C_ receptor mRNA and mbii52, a snoRNA known to regulate RNA editing and RNA splicing of 5-HT_2C_ receptor pre-mRNA, were altered in INI mice compared with wild-type control mice. Moreover, levels of 5-HT_1A_ receptor mRNA were increased in the hippocampus of INI mice. These gene expression changes may underpin the neuroendocrine and behavioural changes observed in INI mice. However, the phenotype of INI mice was not consistent with a globally hyperactive INI receptor encoded by the unedited transcript in the absence of alternate splicing. Hence, the *in vivo* outcome of RNA editing may be neuronal cell type specific.

## Introduction

The 5-hydroxytryptamine_2C_ (5-HT)_2C_ receptor, a G-protein-coupled receptor expressed in the forebrain (Molineaux *et al*., 1989), is implicated in numerous aspects of physiology and behaviour, including appetite regulation and metabolism (Nonogaki *et al*., 1998), anxiety (Heisler *et al*., 2007b), the response to chronic stress (Chou-Green *et al*., 2003) and sleep regulation (Frank *et al*., 2002). Expression of the 5-HT_2C_ receptor, from the encoding *Htr2c* gene, is regulated by circadian signals and the hypothalamo-pituitary-adrenal (HPA) axis (Holmes *et al*., 1995, 1997). Conversely, 5-HT_2C_ receptors may regulate the HPA axis (Heisler *et al*., 2007a). The *Htr2c* pre-mRNA undergoes RNA editing (Burns *et al*., 1997), which results in mRNAs encoding distinct protein sequences (at three amino acids) in the second intracellular loop of the receptor. *In vitro*, the unedited receptor is constitutively active and, as levels of editing increase, the resulting 5-HT_2C_ receptor variants show reduced sensitivity to ligands, reduced basal activity (Niswender *et al*., 1999; Price *et al*., 2001), decreased G-protein coupling (Burns *et al*., 1997) and decreased intracellular signalling (McGrew *et al*., 2004). Furthermore, the constitutively active ‘unedited’ 5-HT_2C_ receptor may be spontaneously internalised in an agonist-independent manner, leaving fewer receptors at the plasma membrane than for ‘edited’ variants (Marion *et al*., 2004; Chanrion *et al*., 2008). Thus, RNA editing appears to be a critical regulation that determines both receptor activity and presence at the membrane. *Htr2c* RNA editing is altered by stress caused by exposure to a water maze (Du *et al*., 2007), early life stress (Bhansali *et al*., 2007) and fluoxetine treatment (Englander *et al*., 2005). Furthermore, levels of *Htr2c* RNA editing can be altered in brains from patients who suffered from schizophrenia (Sodhi *et al*., 2001) or depression (Dracheva *et al*., 2008) and in a murine genetic model of obesity (Schellekens *et al*., 2012). Thus, editing of *Htr2c* pre-mRNA has the potential to significantly impact 5-HT_2C_ receptor signalling in brain, possibly to a greater degree than alterations in levels of gene expression.

Most *in vitro* studies predict that expression of the unedited 5-HT_2C_ isoform would increase 5-hydroxytryptamine (5-HT) signalling, whereas expression of the fully-edited isoform would result in less 5-HT signalling. However, this has only recently been tested *in vivo*. Through a variety of approaches (autoradiography, saturation binding and western blotting) recent studies have shown an increase in total serotonin 2C receptor expression in mice engineered to solely express the fully edited (Valine-Glycine-Valin, VGV) form of the 5-HT_2C_ receptor (Kawahara *et al*., 2008; Morabito *et al*., 2010a; Olaghere da Silva *et al*., 2010). Contrary to expectations, VGV mice fail to thrive at birth and in later life exhibit profound metabolic abnormalities, associated with hypersensitivity to agonists (Kawahara *et al*., 2008; Olaghere da Silva *et al*., 2010). However, mice solely expressing the unedited isoform [Isoleucine-Asparagine-Isoleucine (INI) mice] showed no noticeable metabolic phenotype (Kawahara *et al*., 2008). Moreover, sole expression of the unedited isoform increased anxiety in mice on BALB/c genetic background, but not on C57BL/6 background (Mombereau *et al*., 2010).

Here we describe a distinct line of INI mice, created on a C57BL/6 genetic background, to examine the consequences upon neuroendocrine and behavioural phenotypes associated with dysregulated 5-HT_2C_ receptor signalling. As expression of the unedited INI isoform *in vitro* causes increased alternate splicing of the 5-HT_2C_ receptor to generate a truncated isoform that does not bind receptor (Flomen *et al*., 2004) and prevents full-length transcripts reaching the plasma membrane (Martin *et al*., 2013), the alternate splice site GU1 was also mutated to prevent alternate splicing and reduced receptor function in our INI mice. Furthermore, to test the hypothesis that editing of 5-HT_2C_ receptors is an adaptive or plastic response to inappropriate receptor signalling, we determined the neuroendocrine and behavioural response in INI mice subjected to chronic stress.

## Materials and methods

### Mice

Mice were bred and maintained under standard laboratory conditions in temperature- and humidity-controlled rooms. Food and water were available *ad libitum*, and lights were on from 07:00 to 19:00 h. All animal experiments were approved by the University of Edinburgh Ethical Review Committee and studies were carried out in strict accordance with the UK Home Office Animals (Scientific Procedures) Act, 1986 and the European Communities Council Directive of 24 November 1986 (86/609/EEC). In all experiments, male hemizygous INI mice were tested (*Htr2c* is X-linked). Control mice were wild-type (WT) littermates of INI mice, produced from heterozygous female/hemizygous male matings.

### Generation of INI mice

**Figure 1:**
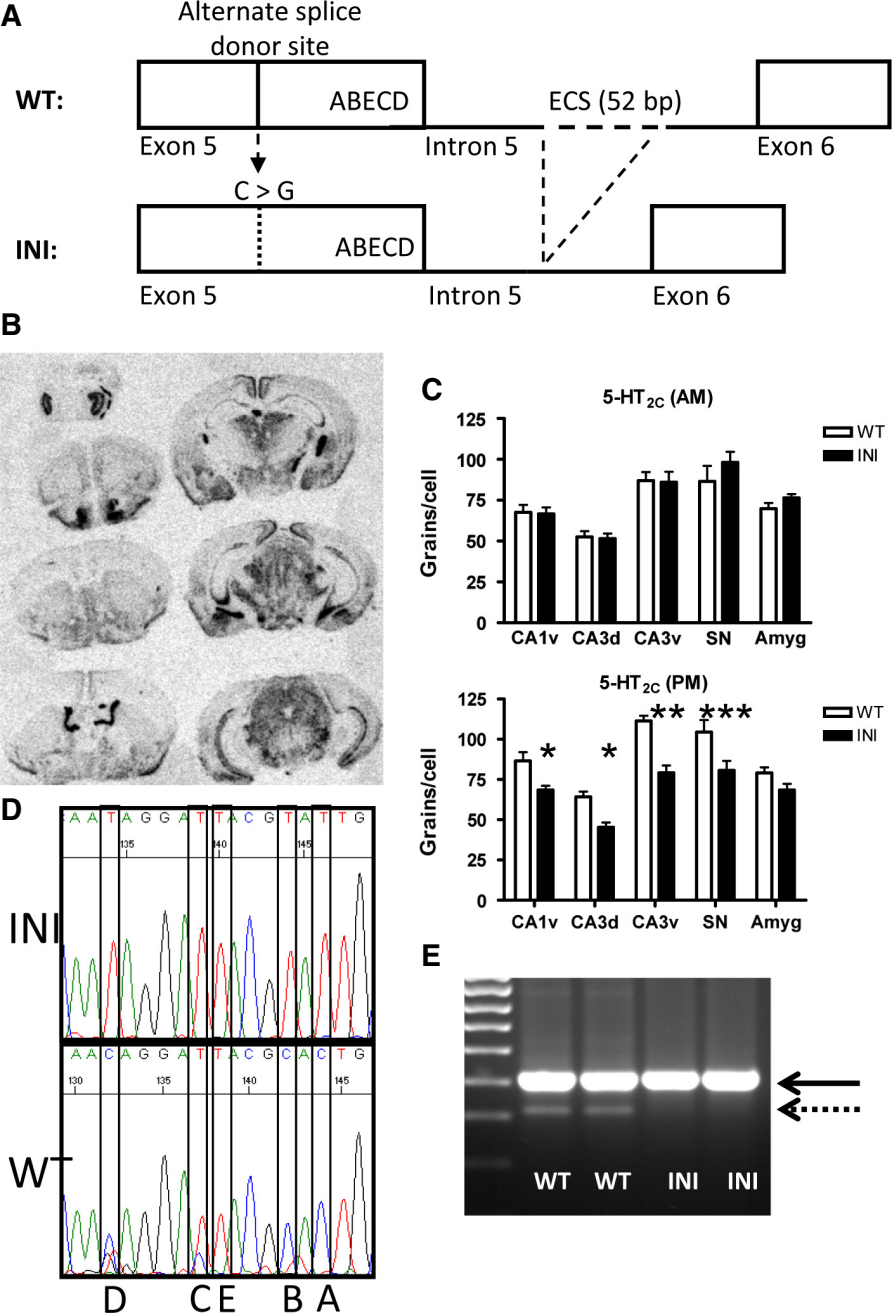
Generation and verification of the INI mouse model. (A) Schematic representation of the targeting strategy used for the INI mice generation. Editing at five sites (A, B, E, C and D) in exon 5 of *Htr2c* was prevented by deleting the exon complementary sequence (ECS) situated in the adjacent intron, thereby inhibiting the formation of a double-stranded RNA structure and the action of the ADAR enzyme (Adenosine Deaminase Acting on RNA). The alternate splice donor site was mutated to prevent the splicing of the transcript. (B) *In situ* hybridisation shows that the brain pattern of INI *Htr2c* RNA expression is normal. (C) Morning and evening levels of *Htr2c* mRNA were quantified from the *in situ* hybridisation; the transcript was differentially expressed in the evening only (*n* = 7–11; *P < 0.05, **P < 0.01, ***P < 0.001). (D) Sequencing traces generated from reverse-transcribed RNA (complementary sequence shown, T and C correspond to an A and G in the *Htr2c* coding sequence) and showing the absence of editing in the INI animals at the five sites (A, B, E, C and D). (E) Following reverse transcription–polymerase chain reaction of *Htr2c* transcripts, this gel shows that the full-length receptor variant is expressed (411 bp, solid line) and the truncated splice variant (dotted line) is missing from the INI mouse RNA (see text for details). SN, Substantia Nigra.

The INI mice were generated by Taconic-Artemis (Germany) by gene targeting in C57BL/6 embryonic stem cells. The targeting strategy is outlined in Fig.1A. Briefly, the *Htr2c* gene was modified to prevent formation of dsRNA and thus RNA editing of the genomic sequence. This was accomplished by removing the exon complementary sequence, which comprises 52 bases in intron 5 (5′-TGGCCATAGAATTGCAGCGGCTATGCTCAATACCTTCGGATTATGTACTGTG-3′). Additionally, to prevent alternate RNA splicing at GU1 [3′ to the editing area in exon 5; nomenclature according to Flomen *et al*. (2004)], which would otherwise result in transcripts encoding a truncated receptor, the GU1 splice donor site cgGtatgta was mutated to cgCtatgta (the point mutation in the splice donor site is indicated in upper case). The sequence resulting from the genetic modifications was verified by DNA sequencing.

Mice were genotyped by polymerase chain reaction on genomic DNA, using primers flanking the exon complementary sequence region of intron 5 (see above), which is deleted in INI mice. The primer sequences were 5′-AAGTGGAAAAGTATGGCTAGTGCAA-3′ and 5′-TGTATCAGTGTTGCCAAAATCCACT-3′, annealing temperature was 62 °C, and the reaction yielded products of 529 bp (WT) or 477 bp (INI). Primers designed to anneal within exon 4 (5′-CAGTAAGCATGGAGAAGAAACTGC-3′) and exon 6 (5′-AGTTCGGGTCATTGAGCACG-3′) were used for the detection of RNA editing in exon 5 through sequencing, as well as for the identification of long and short splice variants.

### Guanosine triphosphate γ S binding assay in membrane fraction of brain

Dissected frozen brain structures (hippocampus and cortex) were homogenised in 20 volumes of cold homogenisation buffer (50 mm Tris-HCl, 3 mm MgCl_2_, 1 mm EGTA, pH 7.4), using 20 strokes of a Dounce homogeniser, on ice. The tissue suspension was centrifuged at 1000 ***g*** for 5 min at 4 °C. The supernatant was then centrifuged at 48 000 ***g*** for 10 min at 4° C. The resulting pellet was resuspended in 200 μL assay buffer (150 mm NaCl, 50 mm Tris-HCl, 3 mm MgCl_2_, 1 mm EGTA, pH 7.4) and frozen at -80 °C. An aliquot was reserved for protein quantification (Bradford assay, Biorad). Binding reactions were carried out in 96-well plates each in a total volume of 200 μL. Protein extracts (10 μg) in binding buffer supplemented with 100 μm guanosine diphosphate (GDP) were pre-incubated for 30 min at 30 °C. Increasing amounts of the 5-HT_2C_ receptor agonist meta-chlorophenylpiperazine (mCPP) (Sigma, UK) were then added with 0.04 nM [^35^S]-guanosine triphosphate γ S (GTPγS) and the reaction incubated for 1 h. Reactions were terminated by filtration through glass fibre membranes (printed Filtermat A, Wallac) using a Combi cell harvester (Skatron) and ice-cold 50 mm Tris-HCl (pH 7.5). Radioactivity (i.e. GTPγS binding to the brain extracts) was measured using MultiLex melt on scintillator sheets (Perkin Elmer) in a liquid scintillation counter (1450 Microbeta Plus; Wallac). Data were normalised by subtracting the unstimulated basal level of binding from the measured increase in radioactivity in response to mCPP. A sigmoidal dose–response equation was applied to the data using prism 4 (GraphPad Software Inc., San Diego, CA, USA) and the regression parameters were used for statistical comparison.

### Behavioural assessment

All procedures were carried out in the morning (08:00–13:00 h). Mice (12–24 weeks old) were moved from the holding room to the behaviour room at 2 h prior to the tests for acclimatisation. Each mouse undertook up to three behavioural tests in random order, except when they were scheduled for the elevated plus maze (EPM) test. The EPM test was always carried out first, as behaviour in this test is sensitive to pre-exposure to other behavioural tests (data not shown).

#### Elevated plus maze

The EPM test was performed as described previously (Holmes *et al*., 2006; Kimura *et al*., 2009). The maze consisted of a Perspex platform in a shape of a plus sign, raised 1 m above the ground. One opposing pair of arms was enclosed by high walls (closed arms) and the other opposing arms were exposed (open arms). Each mouse was placed in the centre of the plus maze, where all of the arms met, and its behaviour was monitored and recorded immediately thereafter for 5 min using a computer tracking system (Limelight, ActiMetrics, IL, USA). The number of open arm entries, time spent in the open arms and the distance travelled within the open arms were measured. Ethological parameters such as stretch attend (stretching out from enclosed arms over the side of the open arm), rearing, grooming, immobility and faeces were scored manually.

#### Open field

The open-field test was performed as described previously (Holmes *et al*., 2006; Kimura *et al*., 2009). In brief, the open field arena consisted of a square box (50 × 50 × 25 cm) divided into 5 × 5 grids, with the central nine squares defined as inner zones and the rest as outer zones. Each mouse was placed in a corner of the box and its activity was monitored and recorded for 5 min using a computer tracking system (Limelight, ActiMetrics). The number of crossings into the inner zones, time spent in the inner zones and the distance travelled within the inner zones were recorded. The experiment was carried out on four consecutive days. The animals were then left for 1 week to rest and retested on day 11.

#### Passive avoidance

Testing took place over two consecutive days, in a two-compartment box (Ugo Basile Biological Research, Comerio, VA, Italy). One side was light (considered to be a more anxiogenic environment) and the other was dark and of equal size, and they were separated by a wall with a sliding door. On day 1, the animals were introduced to the light compartment and, upon opening of the sliding door 90 s later, the latency to enter the dark environment was recorded. On day 2, the animals were introduced into the same light compartment, the latency to change compartment was recorded and they received a light electric shock (0.3 mA) to the feet upon entering the dark side. Eight hours later, the animals were subjected to the same protocol and the latency to enter the dark compartment was recorded (with a maximum test time of 5 min), as an assessment of the decision time for the mouse, a conflict between the anxiogenic light side and potentially noxious dark side.

#### Forced swim test

Mice were placed in clear plastic beakers (26 cm high, 12 cm diameter) filled with tap water (22 °C). The water was renewed between each mouse tested. At 5 s intervals, activity (immobile vs. mobile, and climbing vs. swimming when mobile) was scored by two observers naive to the genotype. All videos were scored a total of four times and the values were averaged.

### Activity measurements

Animals were housed individually in activity cages, with free access to an activity wheel (diameter 23.5 cm). Following an initial acclimation period of 7 days, locomotor activity (wheel revolutions) was measured for a further 7 days, and the data recorded and analysed using the Clocklab software (ActiMetrics).

### 5-Hydroxytryptamine 2C receptor agonist treatment

The 5-HT_2C_ selective agonist (S)-2-(chloro-5-fluoro-indol-l-yl)-1-methylethylamine fumarate (RO 60-0175; Tocris Bioscience, Bristol, UK) was prepared in sterile saline water at 2.5 mg/mL and injected intraperitoneally in mice at a dose of 5 mg/kg. Controls received saline. Mice were then single-housed in a clean cage for 30 min before testing in the open field for 5 min in the morning. Testing was also carried out in the evening using wheel cages. Animals were injected at 30 min before the onset of the dark-phase running activity. Wheel revolutions were monitored for the first 6 h of activity (19:00–01:00 h) on the day prior to testing (basal, following acclimation) and on two subsequent days, following agonist or saline.

### *In situ* hybridisation

Whole brains were removed from mice naive to any treatment, quickly frozen on dry ice and stored at −80 °C. *In situ* mRNA hybridisation was performed as described previously (Holmes *et al*., 1997; Kimura *et al*., 2009). Dried sections were apposed to Hyperfilm β-max film (Kodak) and relative gene expression was assessed by semiquantitative autoradiographic densitometry using MCID basic software 7.0 (InterFocus Imaging Ltd, Linton, UK). Care was taken to ensure all values were within the linear range of the film using ^14^C microscale (Amersham, Chalfont St Giles, UK). Following development of the film, sections were dipped in NTB2 liquid nuclear emulsion (diluted 1 : 1 with distilled water; Anachem, Luton, UK), exposed for 2–4 weeks, photographically processed and counterstained with 1% pyronin Y (Sigma). To quantify the emulsion grains generated by the action of the radioactive probe on the photographic emulsion, a total of two to four areas per brain structure were scored (the background signal was subtracted), and an average score was calculated using the same measurement in the contralateral structure for each brain section. A total of five to seven sections per animal were thus scored and averaged to yield the individual expression value. This work was carried out by an observer naive to the treatments.

Probes were generated from plasmids encoding rat glucocorticoid receptor and corticotrophin-releasing hormone (CRH) (Harris *et al*., 2001) or from plasmids encoding parts of rat 5-HT_1A_ or 5-HT_2A_ (Holmes *et al*., 1995). Two further probe templates were created by cloning polymerase chain reaction amplicons into pGEM T easy (Promega). A mouse dopamine 2 receptor probe (555-1075 of NM_010077.2; primers 5′-TGCCTTCGTGGTCTACTCCT-3′ and 5′-CTTTTCTGGTTTGGCAGGAC-3′) and mouse tryptophan hydroxylase 2 (186-641 of NM_173391.3; primers 5′-TGTCCTTGGATTCTGCTGTG-3′ and 5′-CGTACATGAGGACTCGGTGA-3′) were generated. Another probe template was made by cloning annealed complementary oligonucleotides corresponding to a region of the short snoRNA mbii52 (5′-TCATGAAGAAAGGTGATGACATAAAATTCATGCTCAATAGGATTACGCTA-3′). The resulting plasmids were verified by DNA sequencing.

### Plasma corticosterone measurement

Prior to blood sampling, mice were housed singly for 1 week in a stress-free environment. For the determination of basal morning and evening corticosterone levels, blood samples were taken shortly after the lights were turned on (07:00 h) and off (19:00 h). Mice were killed by decapitation and blood was collected in EDTA-coated tubes (Sarstedt, Germany), centrifuged (10 min, 5000 ***g***) and stored frozen at −80 °C until use. For the determination of corticosterone levels following exposure to chronic restraint stress, animals were housed together (two to three per cage) and on 20 consecutive days mice were inserted into restraint tubes for 10 min. On each of the last 5 days of stress, blood was collected by tail nick into an EDTA-coated tube and processed as for hormone measurement.

Plasma corticosterone levels were measured by radioimmunoassay (Holmes *et al*., 2006) using a polyclonal rabbit anti-corticosterone antibody (kind gift of Dr C. J. Kenyon). The interassay and intra-assay coefficients of variation were <10%.

### Statistical analysis

Data were analysed using prism 4 (GraphPad Software Inc.). Normality was checked, parametric tests were used (t-test, one-way or two-way anova and repeated-measures anova) and statistical significance was set at *P* < 0.05. Due to the heteroscedasticity of the data (between morning vs. evening plasma corticosterone samples), we carried out the statistical analysis of the morning samples with a t-test, simply comparing the effect of genotype on the nadir corticosterone. In some cases (activity wheels and passive avoidance testing), the Grubbs' test was applied (Grubbs, 1969). In this test, an index of data dispersion is calculated for each value x (T_x_=∣(mean-x)/SD∣) and for a given sample size, T_x_ is compared with an appropriate critical value available in the literature (Grubbs, 1969), at the chosen significance threshold of 1%.

## Results

### Generation of INI mice

A schematic representation of the targeting strategy used to generate INI mice is shown in Fig.1A. INI mice were viable, fertile, displayed no gross abnormalities and were born in the expected Mendelian ratio (tested by Chi-squared statistics, not shown). *Htr2c* mRNA was distributed normally in the brains of INI mice killed in the morning (Fig.1B) and at levels similar to WT littermates, in all regions (Fig.1C). Intriguingly, levels of *Htr2c* mRNA were lower in brains of INI mice killed in the evening, compared with WT mice (F_1,70_ = 52.5, *P* < 0.0001) (Fig.1C). Only the unedited form of the receptor was detected by sequencing of cDNA from brains of INI mice (Fig.1D) and alternative RNA splicing from the GU1 site, mutated in INI mice, was abolished (Fig.1E), as predicted from the targeting strategy. As our hypothesis predicted constitutive activity and ligand hypersensitivity of the unedited (INI) receptor, we tested the G-protein coupling and functionality of the 5-HT_2C_ receptors in the INI mouse and investigated the resulting neuroendocrine and behavioural phenotype.

### *In vitro* 5-hydroxytryptamine 2C receptor G-protein coupling is unchanged in INI mice

**Figure 2:**
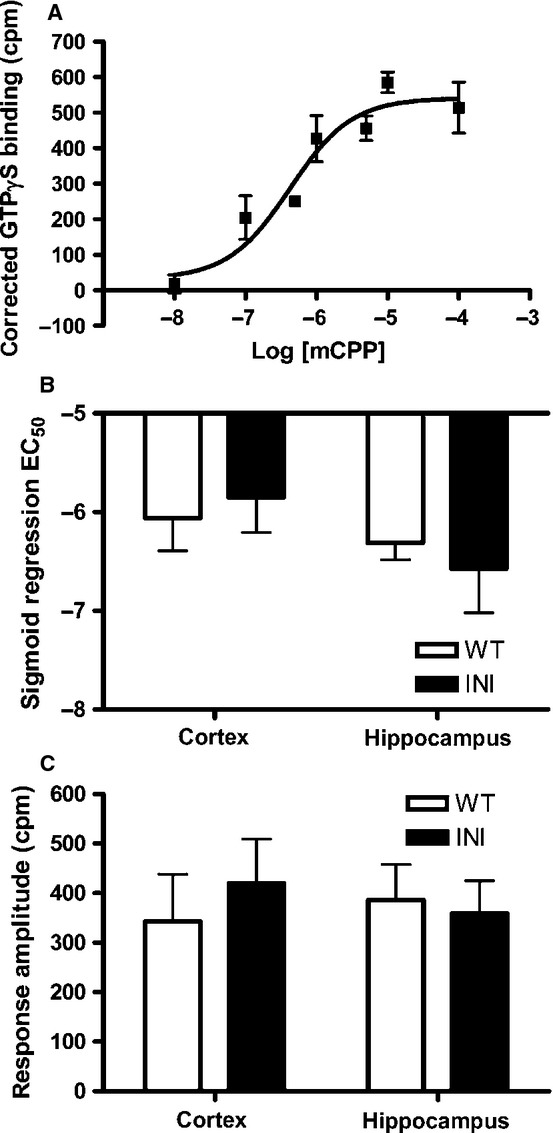
*In vitro* 5-HT_2C_ signalling is not significantly affected in INI mice. (A) Example of sigmoidal dose–response curve for GTPγS binding to hippocampus cell membranes from WT mice using the selective 5-HT_2C_ agonist mCPP. The non-specific ligand binding was subtracted from all values to generate a ‘corrected binding’. Dose–response curves were generated from cell membranes prepared from the cortex and hippocampus; the EC_50_ (B) and response amplitude (C) did not differ between genotypes. Reactions were carried out in triplicate (*n* = 3–5).

To test whether the unedited 5-HT_2C_ receptor was constitutively active and/or hypersensitive to ligand, G-protein coupling to 5-HT_2C_ receptors was determined by GTPγS binding to brain membranes in response to the selective 5-HT_2C_ receptor agonist, mCPP. Membranes from the hippocampus of WT and INI mice showed a sigmoidal dose–response relationship between mCPP concentration and GTPγS binding (as shown in Fig.2A for WT mice). A similar relationship was seen in the cortex of WT and INI mice (not shown). There was no difference in either sensitivity to agonist-induced G-protein coupling (EC_50_) (Fig.2B) or maximum coupling (plateau values) (Fig.2C) between genotypes. Thus, INI and WT mice showed similar 5-HT_2C_ receptor responsivity.

### Daily wheel running activity is similar in wild-type and INI mice

**Figure 3:**
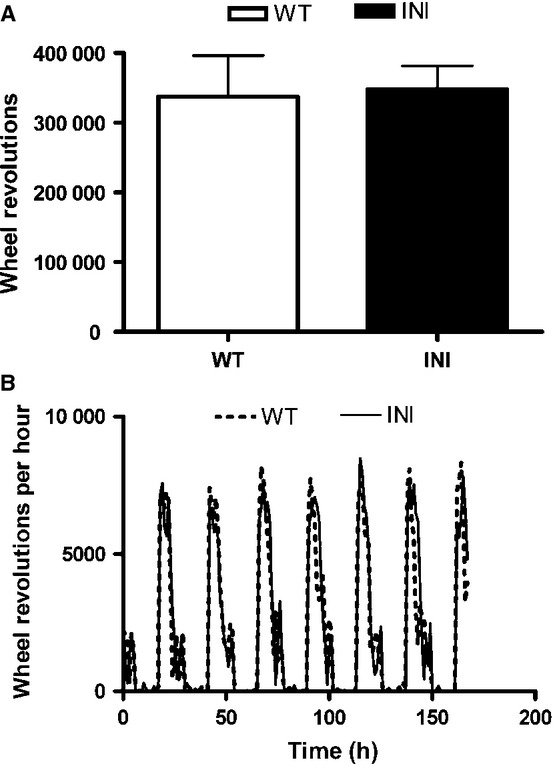
Locomotor activity patterns did not differ between genotypes. Animals (*n* = 7–11) were housed for 1 week in activity cages and their (A) total activity (total number of wheel revolutions; mean+SEM) and (B) daily activity pattern (hourly revolution for each of the 168 h of the week; mean ± SEM) were recorded.

Mice that over-express 5-HT_2C_ receptors in brain are hypoactive (Kimura *et al*., 2009), whereas 5-HT_2C_ receptor-deficient mice are hyperactive (Nonogaki *et al*., 2003). To determine whether INI mice displayed altered overall activity or altered circadian patterns of activity (perhaps reflecting the altered evening levels of *Htr2c* mRNA), we monitored wheel-running behaviour. There was no difference in the total activity (number of wheel revolutions) over the period measured (Fig.3A) or in the circadian pattern of wheel running (Fig.3B), between INI and WT mice.

### Locomotor response to a specific 5-hydroxytryptamine 2C agonist (RO 60-0175) is similar in INI and wild-type mice

**Figure 4:**
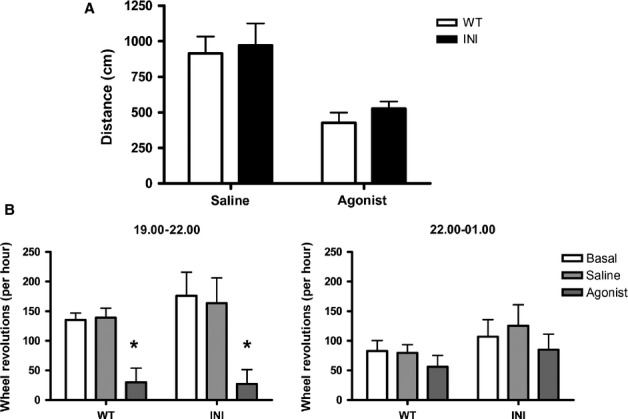
The selective 5-HT_2C_ selective agonist (S)-2-(chloro-5-fluoro-indol-l-yl)-1-methylethylamine fumarate (RO 60-0175) reduced activity in INI and WT mice to a similar extent. (A) The inhibition of locomotion in response to treatment with RO 60-0175 is similar in INI and WT mice tested in the morning. Animals (*n* = 4–6) were injected with 5 mg/kg RO 60-0175 and their locomotor behaviour (total distance travelled) measured in an open field 30 min later. (B) Activity in running wheels was monitored from 19:00 h to 22:00 h and 22:00 h to 01:00 h in mice (*n* = 6) injected with 5 mg/kg RO 60-0175 at 18:30 h, 30 min before the onset of the dark period. The graphs show the hourly wheel revolutions on the day prior to any intervention (basal) and on the day of saline or agonist injection. Values are mean + SEM; *P < 0.05.

The 5-HT_2C_ receptor agonists reduced locomotor activity. Locomotion in the open field was reduced in both INI and WT mice (47 and 54%, respectively, *P* < 0.01 and *P* < 0.05) at 30 min following injection with the selective 5-HT_2C_ receptor agonist, (S)-2-(chloro-5-fluoro-indol-l-yl)-1-methylethylamine fumarate (RO 60-0175; 5 mg/kg) (Fig.4A). The two-way revealed a drug effect (F_1,16_ = 18.53, *P* = 0.0005) but no genotype effect or interaction, suggesting that, at the dose used, the response of INI mice was indistinguishable from that of WT mice. Similarly, injection of RO 60-0175 (5 mg/kg) at 30 min before the onset of wheel-running behaviour decreased activity in both genotypes of mice kept in wheel cages to monitor activity (Fig.4B) but with no difference between genotypes in the 3 h after the onset of the running phase (effect of treatment: F_2,30_ = 12.64, *P* < 0.0001 at 19:00–22:00 h, no significant effect of genotype and no interaction), the time interval when the drug exerted its effects. Therefore, mice that solely expressed unedited 5-HT_2C_ receptors exhibited a similar functional response to a 5-HT_2C_ receptor agonist as WT mice.

### INI mice exhibit a hyperactive hypothalamo-pituitary-adrenal axis

**Figure 5:**
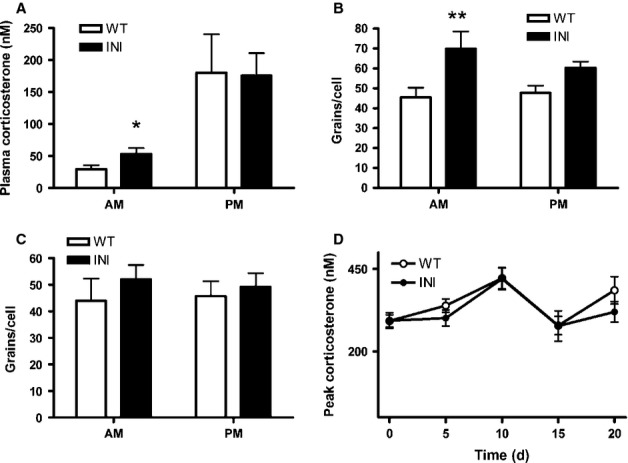
INI mice have an altered HPA axis. (A) Plasma corticosterone (*n* = 11–12) was measured at 07:00 h (lights on) and 19:00 h (lights off). Animals were single housed and kept in a quiet room with minimal disturbance for 5-7 days prior to testing to minimise stress (data compared by t-test). CRH (B) and glucocorticoid receptor (C) mRNA levels were measured in the paraventricular nucleus of the hypothalamus in a subset of the animals from A (*n* = 5–8) by *in situ* hybridisation and silver grains per cell were quantified (data compared by two-way anova followed by Bonferroni testing). (D) Mice were restrained (10 min) daily for 20 days and peak plasma corticosterone was measured at 5 day intervals. The mice did not appear to show any difference in chronic stress adaptation, as measured by stress hormone levels. Values are mean + SEM; *P < 0.05, **P < 0.01.

The 5-HT activation of the HPA axis is mediated in part by activation of 5-HT_2C_ receptors (Heisler *et al*., 2007a). To determine whether sole expression of the INI isoform influenced HPA axis activity, plasma corticosterone levels were measured at the nadir (07:00 h) and peak (19:00 h) of the daily rhythm in glucocorticoids. WT and INI mice both displayed normal rhythmicity (Fig.5A) (F_1,41_ = 15.59, *P* = 0.0003). However, plasma corticosterone levels measured at 07:00 h were higher in INI mice compared with their WT littermates, although peak levels (19:00 h) did not differ (Fig.5A).

The increase in nadir plasma corticosterone levels was accompanied by increased morning levels of mRNA encoding CRH in the paraventricular nucleus of the hypothalamus of INI mice, compared with WT mice (F_1,25_ = 14.41, *P* = 0.0008). However, no differences were observed in the evening (Fig.5B). Therefore, increased CRH drive is likely to underpin the increased nadir plasma corticosterone levels. Levels of glucocorticoid receptor mRNA in the paraventricular nucleus did not differ between INI and WT mice (Fig.5C), suggesting that impaired negative feedback in the paraventricular nucleus is unlikely to account for the elevated morning corticosterone.

#### Corticosterone response to chronic stress was unaltered in INI mice

Evidence suggests that *Htr2c* RNA editing might be a plastic phenomenon, shaped by environmental factors including stress (Du *et al*., 2007). To test the hypothesis that inability to alter *Htr2c* RNA editing and splicing in INI mice reduces adaptation to the effects of chronic stress, mice were subjected to daily restraint for 20 consecutive days. Plasma corticosterone levels were elevated to a similar extent in both genotypes, with no evidence in either of habituation to the stress (Fig.5D). Moreover, weight loss was similar between genotypes (< 10% of initial body weight) over the course of the experiment, with no difference in food intake (not shown).

### INI mice exhibit normal anxiety-type behaviour but reduced depressive-like and fear behaviours

**Figure 6:**
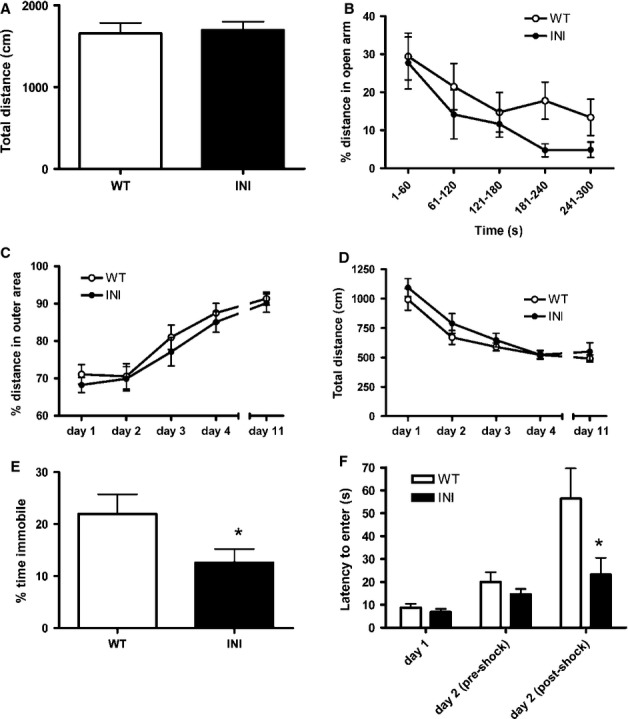
INI mice have an altered mood phenotype. There was no difference in anxiety behaviour between WT (*n* = 17) and INI (*n* = 12) male littermates in the EPM. (A) The total distance covered in the maze over the 5 min test period and (B) the percentage distance in the anxiogenic open arm did not significantly differ between genotypes. Animal locomotion was measured in an open field for four consecutive days, then animals were left to rest for 1 week and tested again (*n* = 14–18). The thigmotaxis (distance travelled in the less anxiogenic outer area) (C) and total distance travelled (D) in the open field did not differ between genotypes. (E) INI mice displayed less learned helplesness in a forced-swim test (*n* = 16 in each group) (t-test comparison, *P < 0.05). (F) Using a passive avoidance test, INI mice exhibited a reduced latency to enter the dark compartment following a mild electric foot shock on day 2, suggesting a lesser memory of a fearful stimulus (two-way anova followed by Bonferroni testing, *P < 0.05). Values are mean ± SEM.

Anxiety-type behaviour was tested in the EPM and open field. The total distance covered in the EPM during the 5 min test period did not differ between WT (1634 ± 121 cm) and INI (1803 ± 143 cm) mice, nor did the percentage distance travelled on the more anxiogenic open arms of the EPM (measured in 60 s time bins) (Fig.6B). Similarly, no differences in anxiety-like behaviour were observed between INI and WT mice in the open field on either the first day of testing or subsequent days (the animals were repeatedly tested to probe the effects of habituation to this environment), measured by the total distance travelled and the percentage distance in the outer zone near the walls (thigmotaxis) (Fig.6C and D).

In contrast to the tests of anxiety-type behaviour, INI mice showed altered depressive-like and fear behaviours. In the forced-swim test, INI mice spent more time swimming and less time immobile than their WT littermates (Fig.6E), indicating lower levels of learned helplessness and thus less depressive-like behaviour than WT mice. In the passive avoidance test of learned fear behaviour, in which mice receive a mild electric shock upon passing from a lit compartment to a dark (preferred) compartment, there was no difference between genotypes in the time taken to enter the dark compartment prior to the shock, but on retesting at 8 h after the electric shock, whereas WT mice showed a delay in re-entry into the dark, INI mice showed no increase in latency to move into the dark compartment (Fig.6E). This suggested a difference in the learned avoidance behaviour, with INI animals exhibiting less or no fearful memory of the shock.

### Investigation of potential compensatory mechanisms in INI mice: gene expression analysis

As the INI mice solely express the unedited form of the 5-HT_2C_ receptor throughout life, there may be compensations within serotonin networks that underpin the observed phenotype. To investigate this, we determined the expression levels of several key genes in serotonin signalling.

**Figure 7:**
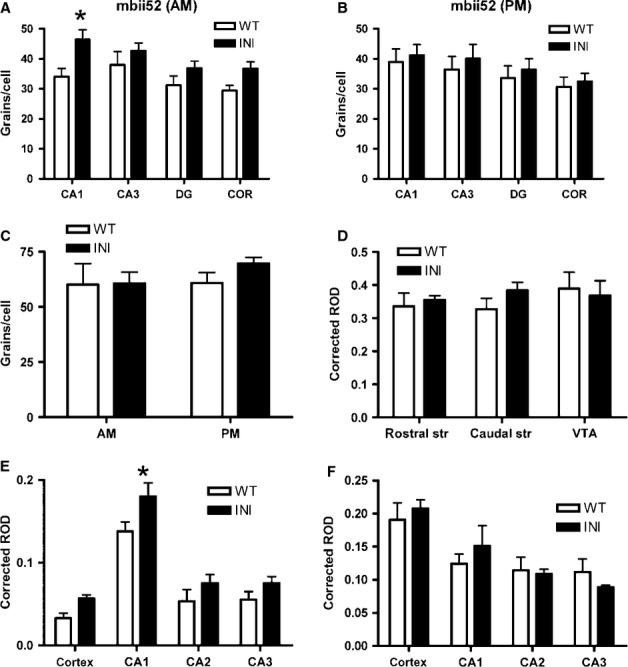
*In situ* hybridisation revealed compensatory gene expression changes in INI mouse brain. mRNA levels were measured by *in situ* hybridisation in INI and WT mice (*n* = 5–8) and silver grains per cell were quantified in brain regions (A-C) in the morning and evening, or signal intensity was quantified by film densitometry (D–F) in the morning samples only. Expression of mbii52 snRNA was increased in INI mice in the morning (A) but not in the evening (B). The brain-specific tryptophan hydroxylase 2 (TPH2), in raphe nuclei, was not altered by the lack of editing (C). The dopamine receptor 2 levels were similar in all regions tested (D). Two other 5-HT receptors were quantified; 5-HT_1A_ levels were higher in INI mice (E) but 5-HT_2A_ levels were not (F). All data were analysed by two-way anova, values are mean + SEM; *P < 0.05. CA, hippocampal cornu ammonis; COR, cortex; DG, hippocampal dentate gyrus; ROD, relative optical density; str, striatum; VTA, ventral tegmental area.

The non-coding transcript, mbii52 (snoRNA), is a known regulator of *Htr2c* RNA editing and splicing (Kishore & Stamm, 2006; Doe *et al*., 2009), hence we investigated whether lack of *Htr2c* RNA editing and splicing impacted upon expression of this transcript in the hippocampus and cortex. Intriguingly, mbii52 was significantly higher in INI mice compared with WT mice, but only in brains sampled in the morning (F_1,44_ = 13.8 *P* < 0.0001) (Fig.7A), not those collected in the evening (Fig.7B). We also determined expression of the rate-limiting enzyme in 5-HT synthesis, tryptophan hydroxylase 2, 5-HT_1A_ receptors and 5-HT_2A_ receptors, as possibly compensatory pathways. Hippocampal 5-HT_1A_ receptor mRNA levels were increased in INI mice, but only in the CA1 subregion (F_1,36_ = 12.3, *P* = 0.0012) (Fig.7E), but there was no change in expression of tryptophan hydroxylase 2 (Fig.7D) or 5-HT_2A_ (Fig.7F) mRNA in any region tested.

As 5-HT signalling through 5-HT_2C_ receptors regulates activity of dopamine neurones, it is plausible that levels of mRNA encoding the D2R receptor, implicated in fear conditioning (Pezze & Feldon, 2004), could be altered in INI mice. However, this was not the case and D2R transcripts were unaltered in the ventral tegmental area or striatum of INI mice compared with WT mice (Fig.7D).

## Discussion

Mice solely expressing the INI isoform of the 5-HT_2C_ receptor from full-length *Htr2c* mRNA, with no editing or alternative splicing, have a hyperactive HPA axis, yet are able to adapt to chronic stress in a similar manner to that observed in WT mice. There are subtle changes in the behaviour and endocrine parameters of INI mice, which are accompanied by gene expression changes within the hippocampus. These data elucidate the complex nature of the consequences of blocking the editing and alternative splicing of the 5-HT_2C_ receptor.

There is a complex interaction between RNA editing and splicing of the 5-HT_2C_ receptor where the INI isoform is associated with increased alternate splicing generating a truncated receptor that is retained in the endoplasmic reticulum and prevents localisation of the full-length receptor at the cell membrane (Flomen *et al*., 2004; Martin *et al*., 2013). To ensure cell membrane localisation of the receptor in our INI model, we also prevented alternate splicing. However, a previous INI model (Kawahara *et al*., 2008) with an intact GU1 site did not exhibit altered levels of the truncated receptor compared with controls, indicating that levels of alternate splicing were not increased by loss of editing. Given that, it must be emphasised that our INI model prevents both editing and alternate splicing of the 5-HT_2C_ receptor.

After confirming the successful generation of a mouse only expressing the unedited isoform of the 5-HT_2C_ receptor (INI mice), we tested whether there was an alteration in G-protein coupling to this receptor. We had anticipated, extrapolating from *in vitro* transfection data, that G-protein coupling and ligand action would be greater in INI mice than in WT mice, which have the majority of their 5-HT_2C_ receptors edited (Burns *et al*., 1997; Price *et al*., 2001). This phenomenon was not recapitulated *in vivo* as agonist-stimulated GTPγS binding and locomotion were similar in INI mice and their controls. However, the failure to see a difference in locomotor response to the RO 60-0175 agonist between genotypes may be due to the dose used. Additionally, if 5-HT_2C_ signalling is altered in the dark phase, in parallel with its gene expression, and not the light phase when the behavioural experiments were carried out, then it will be important to determine the phenotype in the dark phase to confirm the lack of genotype effect.

We had postulated that editing and alternate splicing of the 5-HT_2C_ receptor may be required for the plasticity of the response occurring in chronic adverse environments, given that stressful events have been shown to result in altered editing levels (Englander *et al*., 2005; Du *et al*., 2007). Moreover, 5-HT_2C_ knock-out mice show deficits in habituation and an accentuated response to repeated stress exposure (Chou-Green *et al*., 2003), suggesting a role for the receptor in stress adaptation. However, the neurohormonal and behavioural response to chronic stress was identical in INI and WT mice, suggesting that *Htr2c* RNA editing might not be crucial in the adaptation of the HPA axis to environmental stressors. However, in our experiments, the expected habituation of the corticosterone response to the stress (Herman, 2013) was not observed in either WT or INI mice. We hypothesise that the lack of attenuation in the corticosterone response over the course of the experiment could be due to the intensity of the restraint method that we used. Several groups have reported that the severity of the stressors can prevent such habituation (Kant *et al*., 1983; Pitman *et al*., 1988). Hyperactivity of the HPA axis in INI mice was indicated by their elevated nadir levels of plasma corticosterone and increased morning expression of CRH mRNA in the paraventricular nucleus of the hypothalamus. For the latter measure, there is strong evidence linking CRH mRNA levels to peptide secretion and adrenal gland production of glucocorticoids (Watts, 2005; Aguilera & Liu, 2012). Previous data have suggested a role for the 5-HT_2C_ receptor in HPA axis regulation. In rats, plasma corticosterone levels rise following injection of the 5-HT_2C_ agonist mCPP (Fone *et al*., 1998). In contrast, lack of the 5-HT_2C_ receptor (in knock-out mice) leads to decreased hypothalamic CRH expression and secretion as well as lower plasma corticosterone in response to mCPP (Heisler *et al*., 2007a). Therefore, the HPA hyperactivity could be consistent with increased signalling through the unedited receptor in INI mice.

The disruption of the HPA axis and, more specifically, the profile of plasma corticosterone levels in INI mice are reminiscent of the symptoms of severely depressed humans (de Kloet *et al*., 2007). Sequencing data have suggested an alteration of the *Htr2c* RNA editing patterns in some depressive patients (Dracheva *et al*., 2008) although this observation has not been fully replicated (Zhu *et al*., 2012). We found no evidence of altered anxiety levels in INI mice, which is consistent with data obtained in an independent INI mouse model (Mombereau *et al*., 2010). The genetic background is likely to be an important modifier of anxiety phenotype. On a BALB/c background, INI mice did appear to be more anxious (Mombereau *et al*., 2010), an effect that may be associated with a polymorphism in gene coding for the 5-HT synthetic enzyme tryptophan hydroxylase 2 that results in 50% reduced 5-HT levels in the brain of BALB/c mice compared with C57BL/6 mice (Zhang *et al*., 2004). Although anxiety behaviour was normal, our INI mice exhibited less depressive-like and fear-associated behaviour compared with WT mice (Hackler *et al*., 2007). Interestingly, a 5-HT_2C_ antagonist is presently being promoted as an antidepressant (Millan *et al*., 2011). Furthermore, 5-HT_2C_ activation in the limbic system promotes fear (Campbell & Merchant, 2003) and 5-HT_2C_ antagonism may decrease fear memory (Burghardt *et al*., 2007). Hence, the reduction in depressive-like behaviour and fear memories could be associated with decreased signalling through the INI receptors. This is in contrast to the increased 5-HT_2C_ signalling discussed above as a putative reason for the hyperactive HPA axis.

The HPA axis and behavioural phenotype are consistent with both an increase and decrease of 5-HT_2C_ signalling. Recent findings in the fully edited VGV 5-HT_2C_ receptor mouse (Kawahara *et al*., 2008; Morabito *et al*., 2010b) show that, contrary to the decreased 5-HT_2C_ receptor function anticipated from *in vitro* studies (Niswender *et al*., 1999; Price *et al*., 2001), these mice have increased signalling and sensitivity to the ligand, most probably due to the increased receptor accumulation observed at the plasma membrane. Localisation in the plasma membrane increases with higher levels of editing, via modified interactions with beta arrestin 2, which is responsible for the G-protein-coupled receptor internalisation and trafficking (Marion *et al*., 2004), and through interactions with the truncated form of the 5-HT_2C_ receptor (alternate spliced) retaining the full-length receptor in the endoplasmic reticulum (Martin *et al*., 2013). *In vitro* expression of alternatively edited 5-HT_2C_ variants shows differential G-protein coupling between INI and VGV (Price *et al*., 2001; McGrew *et al*., 2004). The lack of an overall increase or decrease in 5-HT_2C_ function in our mice suggests that the effects of RNA editing and alternate splicing might be affected by the cellular environment in which the RNA is expressed, as this context affects receptor membrane localisation and G-protein expression. Studies addressing the impact of RNA editing and alternate splicing on 5-HT_2C_ signalling in various brain regions and neuronal subtypes may shed light on this discrepancy.

*In vitro* studies show that editing (Vitali *et al*., 2005) and splicing (Kishore & Stamm, 2006) of the *Htr2c* RNA are regulated by the snoRNA mbii52. Mouse models where mbii52 levels were either increased (Nakatani *et al*., 2009) or decreased (Doe *et al*., 2009) express higher levels of *Htr2c* editing. This suggests a dynamic interplay between mbii52 and the *Htr2c* RNA, and we therefore measured snoRNA levels when editing is blocked in our INI mice. The increase that we observed is consistent with this hypothesis. To our knowledge, the only other published data on the regulation of mbii52 are the rise in transcript levels observed during the early phase of memory formation, following in a fear-based learning paradigm (Rogelj *et al*., 2003). The raised expression of the snoRNA in INI mice could impact on other genes not quantified in this study; indeed, mbii52 has been implicated in the splicing of a further five brain transcripts (Kishore *et al*., 2010).

Consistent with previous data (Mombereau *et al*., 2010), C57BL/6 mice with the INI mutation exhibit normal anxiety behaviour, but here we have shown that they have decreased depressive-like behaviour and fear-associated behaviours. Both 5-HT_2C_ receptor agonists and antagonists have been shown to alter affective behaviour, and altered levels of editing of this receptor are associated with both anxiety and depression. The failure to observe altered anxiety in the INI mice suggests that an extra environmental or genetic factor is needed to reveal this phenotype, as environmental manipulations can greatly impact serotonergic and HPA axis signalling (Renoir *et al*., 2013).

Another compensatory gene expression alteration, which we observed when editing was prevented in the INI mice, was an increase in the hippocampal 5-HT_1A_ mRNA levels. This could be correlated with the behavioural findings showing a decrease in fear memory and a lower depressive-like behaviour, in comparison to the WT mice. Transgenic mice lacking the 5-HT_1A_ receptor exhibit more fear memory (Tsetsenis *et al*., 2007), conversely an increase in 5-HT_1A_ levels may logically be associated with less memory of the foot-shock stimulus in our INI mice. There is also pharmacological evidence that treatment of mice with 5-HT_1A_ agonists reduces fear memory (Sigurdsson *et al*., 2007) and increases swimming time in the forced-swim test (Wieland & Lucki, 1990), indicative of a lower depressive-like behaviour. Therefore, at present, we cannot dissociate the compensatory increase in 5-HT_1A_ receptors from a postulated decrease in signalling through 5-HT_2C_ receptors that may underpin the behavioural phenotype observed in INI mice.

In conclusion, we have shown that mice solely expressing the unedited INI form of 5-HT_2C_ receptors exhibit a hyperactive HPA axis driven by increased CRH, a normal response to chronic stress and decreased depressive-like behaviours and fear-associated memory. This neuroendocrine and behavioural phenotype is associated with increased expression of the snoRNA mbii52 and 5-HT_1A_ receptor mRNA. Contrary to expectations, the phenotype of INI mice is not consistent with either a global increase or decrease in 5-HT_2C_ signalling as postulated from *in vitro* studies.

## Abbreviations

5-HT: 5-hydroxytryptamine
CRH: corticotrophin-releasing hormone
EPM: elevated plus maze
GDP: guanosine diphosphate
GTP: guanosine triphosphate
HPA: hypothalamo-pituitary-adrenal
INI: Isoleucine-Asparagine-Isoleucine
mCPP: meta-chlorophenylpiperazine
ROD: relative optical density
VGV: Valine-Glycine-Valine
WT: wild-type
